# Longitudinal Trajectories of Resting Energy Expenditure, Cortisol and IGF-1, and Disease Severity in Critically Ill Patients: A Prospective Pilot Study

**DOI:** 10.3390/nu18142391

**Published:** 2026-07-22

**Authors:** Dimitrios Karayiannis, Anna Elena Yatsulava, Dimitra Katsigianni, Georgios Poupouzas, Charikleia S. Vrettou, Vasileios Issaris, Efthymia Botoula, Marinella Tzanela, Dimitra A. Vassiliadi, Alice G. Vassiliou, Ioanna Dimopoulou

**Affiliations:** 1 Department of Clinical Nutrition, Evangelismos General Hospital, 10676 Athens, Greece; eleanna143g@gmail.com (A.E.Y.); katsigiannidiatrofi@gmail.com (D.K.); 2 Pulmonary Department, Evangelismos General Hospital, 10676 Athens, Greece; poupouzas.gewr.kw@gmail.com (G.P.); vasilisiss@gmail.com (V.I.); 3 Department of Endocrinology, Diabetes and Metabolism, National Expertise Center for Rare Endocrine Diseases, Evangelismos Hospital, 10676 Athens, Greece; vrettou@hotmail.com (C.S.V.); bclf2008@yahoo.gr (E.B.); mtzanel@med.uoa.gr (M.T.);; 4 1st Department of Critical Care Medicine, School of Medicine, National and Kapodistrian University of Athens, Evangelismos Hospital, 10676 Athens, Greece

**Keywords:** resting energy expenditure, indirect calorimetry, insulin-like growth factor-1, cortisol, SOFA score, critical illness, hypermetabolism, pilot study

## Abstract

**Background/Objectives:** Resting energy expenditure (REE) rises during critical illness, but its determinants and prognostic relevance remain incompletely characterized. Identifying a circulating signal that tracks REE could provide a bedside surrogate for metabolic demand where indirect calorimetry is unavailable. This prospective pilot study described longitudinal trajectories of indirect calorimetry-measured REE, Sequential Organ Failure Assessment (SOFA) score, serum cortisol and insulin-like growth factor-1 (IGF-1) over two weeks in the intensive care unit (ICU), examined whether the REE trajectory was attenuated by adjustment for these variables, and generated preliminary effect-size and variance estimates for mortality. **Methods:** This single-center pilot study enrolled 39 critically ill adults at Evangelismos General Hospital (Athens, Greece); sample size was pragmatic, not based on an a priori power calculation. REE (Q-NRG metabolic monitor), cortisol and IGF-1 were measured at admission and days 5–7, 10–11 and 13–14, alongside SOFA. Trajectories were modeled with linear mixed-effects models using all available repeated measures. Confounding was assessed by adding SOFA, cortisol and IGF-1 as covariates and evaluating attenuation of the time effect; no formal mediation analysis was performed. Causes of missing data were quantified and a completers-only sensitivity analysis was undertaken. Mortality was analyzed by Cox regression with ICU length of stay as the time variable. Estimation was emphasized throughout; point estimates and 95% confidence intervals (CIs) are reported in preference to significance testing. **Results:** Mean age was 54.6 ± 18.1 years; 69.2% were male; median admission SOFA was 6 (IQR 3–9). ICU and 28-day mortality were 20.5% (8/39) and 10.3% (4/39). REE/kg rose from 25.3 ± 3.7 to a peak of 27.2 ± 4.2 kcal/kg/day by days 10–11 (likelihood-ratio χ^2^ = 17.2, *p* = 0.0006). Attrition was driven predominantly by discharge alive (18 of 23 patients missing at days 13–14) rather than death (*n* = 2), and the trajectory was preserved in a completers-only sensitivity analysis (χ^2^ = 9.13, *p* = 0.028). Cortisol declined (21.1 to 13.4 μg/dL) and IGF-1 rose (80.0 to 105.4 ng/mL); SOFA was essentially unchanged. REE did not correlate with SOFA, cortisol or IGF-1 at any time-point, and the time effect was not attenuated by adjustment for them. Admission REE was not associated with ICU mortality (HR 1.00, 95% CI 1.00–1.00). Higher admission REE was associated with a longer ICU stay, and this persisted after adjustment for body mass index (*p* = 0.026) and fat-free mass (*p* = 0.001). Age (HR 1.04, 95% CI 1.00–1.09) and admission SOFA (HR 1.18, 95% CI 0.98–1.44) were the covariates most associated with ICU mortality. **Conclusions:** REE rose progressively over the first 10–11 days of critical illness. Its trajectory was not attenuated by adjustment for SOFA, cortisol or IGF-1, which is compatible with—but does not establish—independence from the adrenal and somatotropic markers measured here. Neither REE nor these hormones were associated with mortality; age and disease severity remained the dominant prognostic factors, supporting a larger confirmatory study.

## 1. Introduction

Critical illness is accompanied by a profound metabolic stress response, of which the most prominent and clinically actionable feature is a rise in resting energy expenditure (REE) documented across prospective calorimetry studies in mixed and homogeneous intensive care unit (ICU) populations [1,2]. Indirect calorimetry provides a real-time, bedside estimate of REE from respiratory gas exchange—oxygen consumption and carbon dioxide production—reflecting the oxidative utilization of glucose, fatty acids and amino acids for cellular energy generation [3]. It yields a more valid estimate of metabolic requirements than predictive equations, which do not accurately capture actual energy needs in this population [4,5]; moreover, calorimetry-guided energy delivery has been associated with reduced short-term mortality (risk ratio 0.77) in a meta-analysis of eight randomized trials [6]. Energy expenditure evolves in distinct stages: a modest initial rise during the acute phase—frequently masked by sedation, opioids or neuromuscular blockade—is followed by a more substantial increase that may persist for up to three weeks, a trajectory that informs the recommendation to target 50–70% of measured energy expenditure during the acute phase, as reflected in current ESPEN guidelines [5,7]. That recommendation rests on an incompletely resolved premise: measured energy expenditure reflects the sum of exogenously provided and endogenously produced substrate and, because the latter is rarely quantified at the bedside, any fixed percentage of measured REE is no more validated a nutritional target than a weight-based estimate [8].

The time course of this response has been characterized in several prospective studies. In 98 mechanically ventilated medical patients, Prange et al. reported a stepwise increase in REE during the first week, reaching a quasi-plateau from day 8 onward (19.0 ± 28.7% higher at day 8 and beyond than during days 1–3); body temperature and sedation depth were independently associated with day-to-day REE variability [1]. In the largest study to date, Oosterveld et al. measured 1194 calorimetry readings in 433 patients across seven ICUs and described a biphasic trajectory peaking around day 9–10 before declining; this persisted after adjustment for age, sex, body mass index (BMI), fever, C-reactive protein and renal replacement therapy, and the SOFA score was not associated with the magnitude of REE [2]. A similar non-linear course, peaking around day 5 before plateauing, was reported by Veldsman et al. [9]. Whether hormonal or inflammatory mediators underlie this trajectory was not examined in these studies, leaving open the central question of which physiological drivers account for the rise and subsequent course of REE.

Among the candidate determinants, the neuroendocrine stress response is the most frequently invoked. Critical illness activates the hypothalamic–pituitary–adrenal (HPA) axis and alters peripheral hormone sensitivity, driving a transition from an adaptive catabolic state toward anabolic resistance, across acute, subacute and chronic phases with distinct neuroendocrine, inflammatory and mitochondrial signatures [7,10,11]. Cortisol, the principal effector of the HPA axis, shows an early peak—driven less by increased adrenocorticotropic hormone (ACTH)-stimulated synthesis than by delayed clearance and reduced cortisol-binding proteins—followed by a variable decline [7,11,12]. Adrenocortical secretion may increase up to 20-fold above baseline, with circulating levels reported to correlate with the severity of the insult [13]. The relationship with outcome appears non-linear: in 1412 surgical critically ill patients not receiving corticosteroids, Goder et al. found day-1 cortisol above 50 μg/dL independently associated with 90-day mortality, whereas low concentrations were paradoxically associated with favorable outcomes; the association was most pronounced at low (≤3) or high (≥7) SOFA scores, suggesting that cortisol’s prognostic value is modified by organ dysfunction rather than acting as a linear predictor [14].

The somatotropic axis offers a second candidate signal. Although IGF-1 declines during ICU stay, its relationship with mortality is unclear; some studies report no difference between survivors and non-survivors, whereas septic cohorts show significantly lower IGF-1 in non-survivors, with an inverse correlation with SOFA [15]. Sharshar et al. studied 102 patients who regained consciousness after at least seven days of ventilation and found that in-hospital non-survivors had higher cortisol and lower IGF-1 than survivors, although the number of deaths precluded multivariable adjustment [16]. Cortisol and IGF-1 are therefore biologically plausible correlates of the REE trajectory; whether either is statistically associated with the magnitude of REE or whether these phenomena are temporally coincident but independent at the resolution of clinical sampling has not been well characterized.

Resolving this question has direct clinical consequence. If a measurable circulating signal tracked the magnitude of REE, it could serve as a bedside surrogate for metabolic demand in the majority of ICUs where indirect calorimetry is unavailable, could identify patients whose hypermetabolism is hormonally driven and therefore potentially modifiable, and could inform the timing of nutritional escalation across the acute-to-late phase transition. Conversely, a demonstrated dissociation would imply that energy targets cannot be inferred from adrenal or somatotropic status and that direct measurement remains irreplaceable—a conclusion with practical weight given that calorimetry-guided delivery is associated with lower short-term mortality [6].

Accordingly, the aim of this prospective pilot study was to characterize the longitudinal trajectory of REE during the first two weeks of critical illness and to determine the extent to which its course is associated with disease severity (SOFA) and the neuroendocrine stress response, indexed by cortisol and IGF-1. Specifically, we set out to (i) describe the time course of REE, SOFA, cortisol and IGF-1 across four pre-specified time-points; (ii) test whether the rise in REE is attenuated after adjustment for SOFA, cortisol and IGF-1; and (iii) generate preliminary effect-size and variance estimates to inform an adequately sized confirmatory study. Because no prior study had jointly characterized serial calorimetry with a paired serial adrenal and somatotropic panel, the variance required for a formal a priori power calculation for these endpoints was unavailable, and the sample size was determined pragmatically by feasibility over a fixed enrollment window, consistent with guidance for pilot and feasibility studies [17]. This work is reported explicitly as a pilot study; its mortality analyses are exploratory and hypothesis-generating.

## 2. Materials and Methods

### 2.1. Study Design and Participants

This was a prospective, single-center pilot study conducted in the 1st Intensive Care Unit of Evangelismos Athens General Hospital, a tertiary-care center, between May and November 2024. The unit is a general ICU admitting unselected critically ill patients, including medical, surgical and trauma admissions. Consecutively admitted adults aged 18 years or older expected to remain in the ICU for more than 48 h were screened. Exclusion criteria were brain death; active malignancy; pregnancy; readmission during the same hospital stay or transfer from another ICU; transmissible infections (human immunodeficiency virus and hepatitis); corticosteroid use at ICU admission; terminal illness with expected survival under 48 h; and, owing to the bioelectrical impedance component of the parent protocol, pacemakers or other electronic implants, generalized edema, limb amputation, or pre-existing neuromuscular disease. Patients in whom systemic corticosteroids were initiated during the ICU stay (for example, hydrocortisone for septic shock) contributed all measurements obtained before initiation and were censored from further sampling thereafter; they were not excluded from the analysis, since exogenous glucocorticoids preclude valid interpretation of endogenous cortisol but do not invalidate previously collected data. These criteria are consistent with those applied across our group’s parallel critical-illness cohorts in the same unit [18]. The study was approved by the Institutional Review Board of Evangelismos Hospital (Approval Number 467/11-1-2023) and conducted in accordance with the Declaration of Helsinki. Written informed consent was obtained from the next of kin of all patients.

#### Sample Size and Variance Estimation

No prior study had jointly characterized serial indirect calorimetry with paired serial cortisol and IGF-1 sampling in a mixed ICU population; while serial calorimetry [1,2] and single-time-point hormonal data [16] exist separately, variance estimates for the combined longitudinal design were unavailable. In accordance with guidance for pilot and feasibility studies [17], the sample size (*n* = 39) was determined pragmatically by the recruitment window and by the operational capacity to perform four-time-point calorimetry with concurrent venous sampling using a single trained dietitian, rather than by a pre-specified target effect. A principal deliverable of this pilot is therefore the variance structure required to power a confirmatory study. From the REE/kg mixed model, the between-patient variance was 12.25 (SD 3.50), the within-patient residual variance 1.53 (SD 1.24), and the intraclass correlation 0.89. For reference, with the 19 paired day-1/day-10–11 observations available and the observed within-patient SD, this study had approximately 81% power at α = 0.05 to detect the 1.2 kcal/kg/day within-patient rise observed; 27 paired observations would be required for 80% power to detect a 1.0 kcal/kg/day rise. We make no claim that the mortality analyses were adequately powered; they are explicitly exploratory (Section 4).

This cohort derives from the same protocol (467/11-1-2023) as our companion analysis of phase angle in an overlapping cohort recruited over the same window [19]; the present analysis adds serial hormonal measurements and survival linkage not examined in that report. A methodologically distinct study from the same unit examined glucocorticoid receptor isoform expression [18]; it was conducted under a separate approval (444–28/09/2023) with a different enrollment period and therefore represents a parallel, non-overlapping cohort.

### 2.2. Measurements and Outcome Definitions

Indirect calorimetry was performed by a trained clinical dietitian using the Q-NRG metabolic monitor (COSMED, Rome, Italy) in canopy or ventilator-coupled mode at four pre-specified time-points: day 1 (ICU admission), days 5–7, days 10–11 and days 13–14. REE was recorded in kcal/day and normalized to actual body weight (REE/kg). Concurrently, venous samples were drawn for serum cortisol (μg/dL) and IGF-1 (ng/mL); cortisol was measured by chemiluminescent immunoassay on an Immulite 2000 system (Siemens Healthineers, Forchheim, Germany) [18]. Total, not free cortisol and total, not free IGF-1 were measured; ACTH, cortisol-binding globulin, IGFBP-3, catecholamines, thyroid hormones and inflammatory cytokines were not assayed. SOFA was recorded at each time-point. Height, weight, BMI and bioelectrical-impedance-derived fat-free mass (FFM) were recorded at admission. Invasive mechanical ventilation (MV) status at admission and total duration of MV were recorded.

Outcomes were defined a priori as follows. ICU mortality was death from any cause before ICU discharge, irrespective of timing. A 28-day mortality was death from any cause within 28 days of ICU admission. ICU length of stay (LOS) was used as the time-to-event variable in survival analyses, with ICU death as the event and discharge alive as a censoring event. Every 28-day death in this cohort was also an ICU death; there were no post-discharge deaths within 28 days.

### 2.3. Statistical Analysis

Continuous variables are reported as mean ± SD or median (IQR). Longitudinal trajectories were modeled using linear mixed-effects models with a random intercept per patient and time-point as a categorical fixed effect (reference: day 1), using all available repeated measures rather than restricting to complete cases. Overall time effects were assessed by likelihood-ratio test against an intercept-only model. Cross-sectional associations between REE/kg and SOFA and cortisol and IGF-1 were assessed by Spearman correlation at each time-point. To assess whether these variables confounded the REE trajectory, they were added as fixed-effect covariates to the mixed model for REE/kg and the time effect was re-examined for attenuation. We emphasize that this constitutes covariate adjustment, not a formal mediation analysis: no decomposition of direct and indirect effects and no estimation of an indirect effect with confidence intervals was performed, and no causal mediation is claimed.

Missing data were characterized descriptively by cause at each time-point. Because the missing-at-random (MAR) assumption underlying available-case mixed-model estimation cannot be verified empirically from observed data [20], we did not attempt to test it; instead, we (i) quantified the causes of missingness, (ii) compared completers with non-completers on admission variables, and (iii) refitted the REE/kg trajectory model restricted to completers as a sensitivity analysis. Between-group comparisons used the Mann–Whitney U test and Fisher’s exact test. Associations of admission variables with LOS and MV duration were assessed by Spearman correlation; because absolute REE scales with body size, the association between admission REE and LOS was additionally examined in linear models of log(LOS) adjusted for BMI and, separately, for FFM.

Cox proportional hazards regression, with ICU LOS as the time variable, was used to estimate univariable and multivariable associations with ICU mortality and univariable associations with 28-day mortality. Given the limited number of events (8 ICU deaths; 4 28-day deaths), multivariable models were restricted to two covariates and proportional hazards were checked using Schoenfeld-residual tests. The duration of MV was not entered into any survival model; it accrues over the ICU stay and is strongly collinear with ICU LOS (Spearman ρ = 0.80), which constitutes the model’s time axis, so any hazard ratio for it would be uninterpretable. For the same reason, MV duration was not compared between survivors and non-survivors, since a patient who dies early cannot accrue ventilator days. MV duration is therefore reported descriptively and as an outcome of the clinical course only.

Consistent with the exploratory, estimation-focused nature of a pilot study, point estimates and 95% confidence intervals are reported in preference to significance testing; *p*-values are provided as continuous descriptive quantities and are not interpreted against a binary threshold, and the language of statistical significance has been avoided. Analyses were performed in Python 3.12 (statsmodels, lifelines, SciPy). The pilot and feasibility intent and the emphasis on estimation were established a priori. Reporting follows the STROBE recommendations for cohort studies [21].

## 3. Results

### 3.1. Patient Characteristics

Thirty-nine patients were included. Mean age was 54.6 ± 18.1 years, 27 (69.2%) were male, and mean BMI was 26.9 ± 3.1 kg/m^2^. Median admission SOFA was 6 (IQR 3–9) and median ICU LOS was 12 days (IQR 7–34). Twenty-five patients (64.1%) were mechanically ventilated at admission; median MV duration was 10 days (IQR 0–22). ICU mortality occurred in eight patients (20.5%) and 28-day mortality in four (10.3%). Of the eight ICU deaths, four occurred within 28 days (ICU days 10, 12, 14 and 19) and four occurred at or beyond day 28 (ICU days 28, 37, 40 and 42); the latter were long-stay, persistently critically ill patients who died late and therefore contribute to ICU but not to 28-day mortality. This accounts for the difference between the two mortality figures and reflects the long right tail of ICU stay in this cohort (maximum 70 days) rather than a definitional inconsistency; no death followed a documented transition to comfort care. Baseline characteristics are summarized in Table 1.

### 3.2. Attrition, Missing Data and Sensitivity Analysis

Because attrition across time-points was substantial (39, 28, 19 and 16 patients at the four respective time-points), we quantified its causes (Table 2). Missingness was driven predominantly by discharge alive rather than by death; of the 23 patients without a day-13–14 measurement, 18 had been discharged alive, only 2 had died, and 3 remained in the ICU without a measurement. Consequently, the patients retained at the later time-points were those who remained critically ill, and comparison of completers (*n* = 16) with non-completers (*n* = 23) confirms this direction; completers had a higher admission REE (median 2310 vs. 1856 kcal/day, *p* < 0.001), a longer ICU stay (38.5 vs. 8.0 days, *p* < 0.001) and a higher admission SOFA score (7.5 vs. 5.0, *p* = 0.054), while ICU mortality did not differ between the groups (4/16 vs. 4/23, *p* = 0.694). Any informative-dropout bias in this cohort therefore operates toward sicker, longer-staying patients at later time-points, not toward healthier survivors. In a sensitivity analysis restricted to completers, the REE/kg trajectory was preserved with a similar magnitude (likelihood-ratio χ^2^ = 9.13, *p* = 0.028; +1.05 kcal/kg/day at days 5–7 and +1.10 kcal/kg/day at days 10–11 versus day 1, compared with +1.26 and +1.21 in the full available-case analysis), indicating that the observed rise is not an artefact of differential dropout.

### 3.3. Longitudinal Trajectories of REE, SOFA and Hormonal Parameters

**Figure 1:**
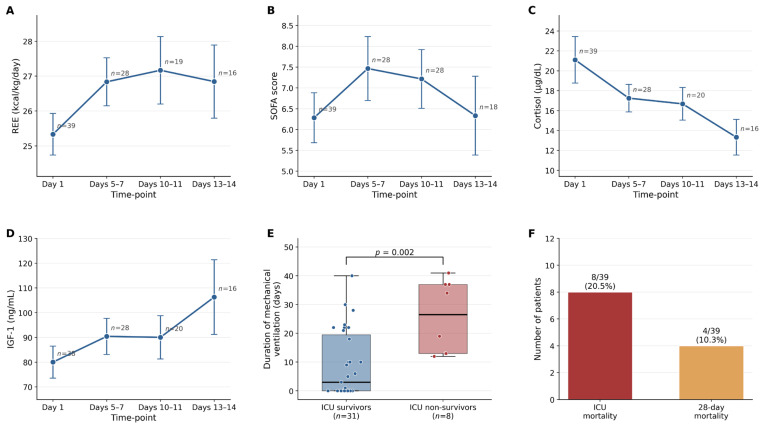
Longitudinal trajectories and outcomes in 39 critically ill patients. (**A**) REE normalized to body weight; (**B**) SOFA score; (**C**) serum cortisol; (**D**) serum IGF-1, each across four ICU time-points. Data are mean ± standard error of the mean (SEM); the number of patients contributing at each time-point is annotated. (**E**) Duration of mechanical ventilation by ICU outcome, shown as a box-and-whisker plot (median, interquartile range, whiskers to 1.5 × IQR) with individual data points overlaid; the *p*-value is from a Mann–Whitney U test. This comparison is descriptive only and is confounded by in-ICU time at risk (Section 2.3 and Section 4). (**F**) Number of ICU and 28-day deaths in the cohort. ICU, intensive care unit; IGF-1, insulin-like growth factor-1; IQR, interquartile range; REE, resting energy expenditure; SOFA, Sequential Organ Failure Assessment.

REE rose over the four time-points (likelihood-ratio χ^2^ = 13.4, *p* = 0.0038), from 2051 ± 490 kcal/day at admission to 2237 ± 498 at days 5–7 and 2378 ± 528 at days 10–11, before declining numerically to 2345 ± 500 at days 13–14. The same pattern was evident for REE/kg (χ^2^ = 17.2, *p* = 0.0006), which rose from 25.3 ± 3.7 to 26.8 ± 3.6 kcal/kg/day by days 5–7 (+1.26, 95% CI 0.59–1.93) and 27.2 ± 4.2 by days 10–11 (+1.21, 95% CI 0.46–1.96), without further change at days 13–14. The SOFA score was essentially unchanged across time-points (6.3 ± 3.8 to 7.3 ± 3.4; χ^2^ = 3.9, *p* = 0.277). Cortisol declined progressively from 21.1 ± 14.6 μg/dL to 13.4 ± 7.4 μg/dL at days 13–14 (−7.9, 95% CI −13.8 to −2.0), and IGF-1 rose from 80.0 ± 40.0 to 105.4 ± 62.5 ng/mL (+24.6, 95% CI 6.8–42.4). These trajectories are summarized in Table 3 and Figure 1.

### 3.4. Cross-Sectional Associations and Covariate Adjustment

At no time-point did REE/kg correlate with SOFA, cortisol or IGF-1 (all |ρ| ≤ 0.32; admission: SOFA ρ = −0.003; cortisol ρ = 0.14; IGF-1 ρ = 0.23). When SOFA, cortisol and IGF-1 were added simultaneously as covariates to the mixed model for REE/kg, the time effect was not materially attenuated (time-point coefficient 0.238 in the base model versus 0.233 after adjustment), and none of the covariates was appreciably associated with REE/kg. The REE trajectory is therefore not attenuated by adjustment for these variables. We emphasize that this is a confounding assessment, not a mediation analysis; it is compatible with independence between the hypermetabolic response and the measured hormonal profile, but it does not establish it and it cannot exclude a relationship operating on a shorter timescale than our sampling interval or through mediators we did not measure (Section 4).

### 3.5. Admission Variables and Mortality

Patients who died in the ICU were older (median 66.0 vs. 54.0 years) and had higher admission SOFA scores (9.0 vs. 6.0) than survivors. Admission REE, REE/kg, cortisol and IGF-1 did not differ appreciably between ICU survivors and non-survivors (Table 4). In accordance with the collinearity considerations set out in Section 2.3, the duration of mechanical ventilation is not compared between survivors and non-survivors, since such a comparison is confounded by in-ICU time at risk.

### 3.6. Cox Proportional Hazards Analysis

In univariable Cox regression for ICU mortality, age was the covariate most associated with the hazard (HR 1.04 per year, 95% CI 1.00–1.09) and admission SOFA showed a positive but imprecise association (HR 1.18 per point, 95% CI 0.98–1.44). Neither admission REE (HR 1.00, 95% CI 1.00–1.00) nor REE/kg (HR 0.88, 95% CI 0.73–1.07) was associated with ICU mortality, and confidence intervals for cortisol (HR 1.01, 95% CI 0.96–1.05) and IGF-1 (HR 0.99, 95% CI 0.97–1.01) were centered close to unity. Univariable estimates for 28-day mortality are reported alongside for consistency; with only four events, all estimates are extremely imprecise, and none excludes the null (Table 5). These are presented for completeness and for potential future meta-analytic use rather than for inference. In a parsimonious multivariable model combining REE/kg and admission SOFA, proportional hazards were satisfied (Schoenfeld *p* > 0.45) and the concordance index was 0.74; SOFA was associated with ICU mortality (HR 1.25, 95% CI 1.00–1.57) and REE/kg was not (HR 0.85, 95% CI 0.69–1.04). In accordance with Section 2.3, no hazard ratio is reported for the duration of mechanical ventilation.

### 3.7. Baseline Predictors of Prolonged ICU Stay and Mechanical Ventilation

We next examined whether admission variables were associated with the eventual length of ICU stay and duration of MV. Because both are continuous features of the clinical course, correlations were assessed on the continuous scale (Table 6). Admission REE and admission SOFA were each moderately associated with a longer ICU stay (REE ρ = 0.55; SOFA ρ = 0.43) and a longer duration of MV (REE ρ = 0.47; SOFA ρ = 0.53), whereas age, REE/kg, cortisol and IGF-1 were not. Because absolute REE scales with body size, we tested whether this association merely reflected larger patients. It did not; the association between admission REE and ICU length of stay persisted after adjustment for BMI (β = 0.00088 per kcal/day, *p* = 0.026) and after adjustment for FFM (β = 0.00117, *p* = 0.001); REE normalized to FFM remained associated with length of stay (ρ = 0.56); and FFM alone was not associated with length of stay (ρ = 0.02). These analyses are interpreted in the forward direction only—higher baseline severity and a higher baseline absolute metabolic rate mark patients who subsequently experience a longer, more ventilation-dependent course—and not as statements about the prognostic value of the course variables themselves.

## 4. Discussion

This prospective pilot study characterized the joint longitudinal trajectories of indirect calorimetry-measured REE, SOFA score, cortisol and IGF-1 over the first two weeks of critical illness. Three findings merit discussion. First, REE rose progressively, replicating prior serial calorimetry studies [1,2,7,9]; this trajectory analysis used all available repeated measures, was robust to a completers-only sensitivity analysis, and is the most secure finding of the study. Second, despite the temporal co-occurrence of a falling cortisol and a rising IGF-1 trajectory, neither hormone nor SOFA correlated with the magnitude of REE at any time-point, and the time effect was not attenuated by adjustment for them. Third, neither REE nor these hormones was associated with ICU or 28-day mortality, while age and organ-dysfunction severity carried the prognostic signal.

The dissociation between the REE trajectory and the SOFA/hormonal trajectories is an informative negative finding, though its scope must be stated carefully. It indicates that the rise in measured energy expenditure is not attenuated by adjustment for total cortisol, total IGF-1 or SOFA at a sampling density of four time-points over two weeks. It does not establish that the hypermetabolic response is independent of the neuroendocrine stress response in general; we did not measure ACTH, free cortisol, cortisol-binding globulin, IGFBP-3, catecholamines, thyroid hormones or inflammatory cytokines, and our data are silent on their contribution. The magnitude of the rise we observed (approximately 1.2 kcal/kg/day, or roughly 5%, by days 10–11) is more modest than the 19.0% increase by day 8 reported by Prange et al. [1], although both studies converge on a plateau emerging toward the end of the first week to 10 days. This is consistent with the larger cohort of Oosterveld et al., in which REE peaked around day 9–10 before declining [2], and with Veldsman et al., who observed a peak around day 5 followed by a plateau [9]. Importantly, our null finding for SOFA is not idiosyncratic: Oosterveld et al. found no association between SOFA and the magnitude of REE in a cohort roughly tenfold larger [2], and Veldsman et al. found measured energy expenditure to be associated with age, sex and BMI but not with SOFA, APACHE II or C-reactive protein [9]. Two independent cohorts therefore corroborate the dissociation between organ-dysfunction severity and metabolic rate, which makes it unlikely that our result is solely an artefact of limited power.

Several determinants of REE that we did not measure are plausible contributors to the trajectory and to its unexplained variance. Prange et al. identified body temperature and sedation depth as independent predictors of day-to-day REE variability [1]; neither was captured here, nor was nutritional intake, propofol administration or the inflammatory state. Inflammatory status in particular is a leading candidate, since cytokine-mediated thermogenesis is a recognized driver of hypermetabolism, and a cytokine panel is a priority for the confirmatory study we propose. This is consistent with a conceptual framework in which REE is driven by a composite of catecholamine signaling, cytokine-mediated thermogenesis, work of breathing, fever, sedation depth and substrate mobilization, of which the adrenal axis is only one—and not necessarily rate-limiting—component [7,10,11]. Recent appraisals argue that the catabolic response of critical illness is an evolutionarily conserved, largely nutrition-independent program rather than a state correctable by circulating hormone concentrations alone [8], and narrative reviews have proposed integrating biomarkers and metabolomic signatures to define clinical endotypes that may better account for its heterogeneity [7]. It also remains possible that the relevant biological window for hormone–REE coupling is shorter than our sampling interval or that the study was simply too small to detect modest correlations; detecting a correlation of ρ = 0.3 with 80% power at α = 0.05 would require approximately 85 paired observations, more than double what was available at any single time-point.

The decline in cortisol is consistent with resolution of the acute stress response in a cohort of moderate severity, although it must be interpreted cautiously given that corticosteroid use at admission was an exclusion criterion. Mechanistically, the elevated cortisol of the acute phase arises predominantly from reduced clearance and a fall in cortisol-binding globulin rather than increased ACTH-driven output [7]; because we measured neither ACTH nor binding proteins nor free cortisol, we cannot determine whether the observed decline reflects falling adrenal output, recovering binding-protein synthesis, or both. Our cohort’s admission cortisol (21.1 ± 14.6 μg/dL) lies well below the >50 μg/dL threshold at which Goder et al. observed an independent association with 90-day mortality [14], which may explain why we detected no cortisol–mortality association: a continuous-variable Cox model is poorly suited to detect a threshold effect, and few of our patients reached the range in which that relationship was apparent.

The rise in IGF-1 is compatible with recovery of hepatic anabolic signaling as the acute phase resolves. An important alternative explanation, however, is nutritional: IGF-1 is nutritionally regulated and falls with caloric and protein deficit, so a rise coinciding with the progressive establishment of enteral and parenteral nutrition may reflect improving nutritional delivery rather than genuine anabolic recovery. Because we did not systematically record delivered energy and protein, we cannot distinguish these possibilities; nor, having measured total rather than free IGF-1 or IGFBP-3, can we comment on the bioavailable fraction. Concurrent capture of nutritional intake is therefore essential in any confirmatory study.

Admission REE was not associated with mortality, and this null result should not be over-interpreted; with eight ICU deaths, the confidence intervals remain compatible with clinically meaningful effects in either direction. Beyond mortality, the clearest forward-looking signal was that higher admission SOFA and higher absolute admission REE marked patients who subsequently experienced a longer, more ventilation-dependent course. That this association is not merely a function of body size is supported by its persistence after adjustment for BMI and fat-free mass and by the observation that fat-free mass alone did not predict length of stay. A plausible interpretation is that absolute REE captures the total metabolic burden of the illness—the aggregate of metabolically active mass and the intensity of the stress response—whereas weight-normalized REE is diluted by adiposity and by fluid accumulation, both of which inflate the denominator without contributing proportionally to energy expenditure. This is consistent with the observation of Veldsman et al. that measured energy expenditure is associated with body size and composition variables rather than with severity scores [9]. Because length of stay and ventilation duration are themselves downstream features of the clinical course, we interpret this only in the forward direction and make no claim that either course variable is prognostic in its own right.

### Strengths and Limitations

The principal strength of this study is that it is, to our knowledge, among the first prospective cohorts to pair serial indirect calorimetry with serial cortisol and IGF-1 sampling at fixed, calendar-based time-points across the first two weeks of critical illness, generating trajectory and variance data not previously available. The mixed-effects models use all available repeated measures, preserving power and limiting selection bias, and the robustness of the principal finding was formally examined in a completers-only sensitivity analysis.

Its limitations follow largely from the pilot design and define the scope of inference. The sample size (*n* = 39) and event rate (eight ICU and four 28-day deaths) were pragmatic rather than powered, so all mortality analyses are hypothesis-generating. Using Schoenfeld’s formula [22], and assuming two-sided α = 0.05, 80% power and a conservative standardized hazard ratio of 1.5 per 1-SD increase (deliberately more conservative than the estimates observed here, which are liable to inflation), a confirmatory study would require approximately 48 ICU deaths, corresponding to roughly 235–320 patients at an ICU mortality of 15–20%. Attrition was substantial (39 to 16) and, although we have shown that it was driven predominantly by discharge alive rather than death, that completers were sicker rather than healthier, and that the trajectory is preserved among completers, the missing-at-random assumption underlying available-case estimation cannot be verified empirically [20]; our sensitivity analysis supports robustness but cannot prove it, and a pattern-mixture or shared-parameter joint model would be required to interrogate a not-missing-at-random mechanism directly. We did not fit a joint longitudinal–survival model because, with 39 patients and eight events, it would be substantially over-parameterized and would convey false precision. A further limitation of the design is temporal resolution: four time-points over 14 days cannot capture hour-to-hour or day-to-day covariation between cortisol and metabolic rate, and a null association at this resolution is evidence about the resolution, not about the biology. ICU LOS served as the Cox time variable; because MV duration is strongly collinear with it (ρ = 0.80), MV could not be evaluated as a prognostic variable and is reported descriptively only. We did not measure ACTH, free cortisol, cortisol-binding globulin, IGFBP-3, catecholamines, thyroid hormones or cytokines, nor markers of protein catabolism or endogenous substrate production [5,8], nor body temperature, sedation depth or nutritional intake [1]. Finally, the exclusion criteria create a selected cohort: excluding patients receiving corticosteroids at admission selects against septic shock and critical-illness-related corticosteroid insufficiency—precisely the subgroup in which the adrenal axis is most perturbed and a cortisol–REE relationship might be most detectable—so our null hormonal findings may not generalize to steroid-treated or predominantly septic populations. The further exclusion of malignancy, transmissible infection, implants and amputation and a cohort of moderate severity (64% ventilated, median SOFA 6) likewise limit generalizability to unselected ICU populations.

## 5. Conclusions

In this prospective pilot study of 39 critically ill patients, REE rose over the first 10–11 days of ICU stay, in parallel with—but not attenuated by adjustment for—concurrent trajectories of SOFA score, cortisol and IGF-1. This is compatible with, but does not establish, independence of the hypermetabolic response from the adrenal and somatotropic markers measured here; it cannot be generalized to hormonal or inflammatory mediators we did not assay. Neither admission REE nor these hormones was associated with ICU or 28-day mortality, while age and disease severity carried the prognostic signal. Higher absolute admission REE and higher admission SOFA marked patients destined for a longer, more ventilation-dependent course, and this was not explained by body size. These findings argue against a simple, direct coupling between circulating adrenal hormone concentrations and the magnitude of the hypermetabolic response, and they support the continued use of direct measurement rather than hormonal or severity-based inference of energy requirements. Consistent with its design, this work provides the trajectory data, variance components and sample-size projections needed to plan an adequately powered confirmatory study with denser hormonal sampling, concurrent capture of nutritional intake and inflammatory markers, and joint longitudinal–survival modeling.

## Figures and Tables

**Table 1:** Baseline characteristics of the study population (*n* = 39).

Variable	Value
Age, years	54.6 ± 18.1
Male sex, *n* (%)	27 (69.2%)
Body mass index, kg/m^2^	26.9 ± 3.1
Fat-free mass, kg	39.6 ± 4.9
SOFA score at admission, median (IQR)	6 (3–9)
ICU length of stay, days, median (IQR)	12 (7–34)
Mechanical ventilation at admission, *n* (%)	25 (64.1%)
Duration of mechanical ventilation, days, median (IQR)	10 (0–22)
REE at admission, kcal/day	2051 ± 490
REE/kg at admission, kcal/kg/day	25.3 ± 3.7
Cortisol at admission, μg/dL	21.1 ± 14.6
IGF-1 at admission, ng/mL	80.0 ± 40.0
ICU mortality, *n* (%)	8 (20.5%)
28-day mortality, *n* (%)	4 (10.3%)

Data are mean ± SD, median (IQR), or *n* (%). Duration of mechanical ventilation is reported descriptively and is not used as a predictor in any survival model (Section 2.3). ICU, intensive care unit; IGF-1, insulin-like growth factor-1; IQR, interquartile range; REE, resting energy expenditure; SD, standard deviation; SOFA, Sequential Organ Failure Assessment.

**Table 2:** Causes of missing data at each time-point, comparison of completers with non-completers, and sensitivity analysis of the REE/kg trajectory.

Time-Point	Measured, *n*	Discharged Alive	Died	In ICU, No Measurement	Total Missing
Day 1	39	0	0	0	0
Days 5–7	28	7	0	4	11
Days 10–11	19	13	0	7	20
Days 13–14	16	18	2	3	23
**Admission Variable**	**Completers (*n* = 16)**		**Non-Completers (*n* = 23)**		***p***
Age, years	56.0 (42.0–70.8)		58.0 (53.2–63.8)		0.801
SOFA score	7.5 (5.8–9.2)		5.0 (2.0–9.0)		0.054
REE, kcal/day	2310 (2014–2543)		1856 (1568–2040)		<0.001
REE/kg, kcal/kg/day	26.2 (23.0–30.0)		23.9 (22.4–26.9)		0.149
Cortisol, μg/dL	24.4 (15.4–33.7)		17.3 (8.2–25.6)		0.184
IGF-1, ng/mL	85.0 (60.8–112.5)		75.5 (50.0–98.0)		0.375
ICU length of stay, days	38.5 (26.8–44.8)		8.0 (4.0–11.0)		<0.001
ICU mortality, *n* (%)	4 (25.0%)		4 (17.4%)		0.694
**REE/kg Trajectory Model**	**LR χ^2^ (*p*)**		**Rise vs. Day 1 (Days 5–7/Days 10–11)**		
All available cases (*n* = 39)	17.24 (0.0006)		+1.26/+1.21 kcal/kg/day		
Completers only (*n* = 16)	9.13 (0.028)		+1.05/+1.10 kcal/kg/day		

Completers are patients with a day-13–14 measurement. Between-group comparisons used the Mann–Whitney U test (continuous) and Fisher’s exact test (categorical); values are median (IQR). The missing-at-random assumption cannot be tested empirically; the completers-only model is presented as a sensitivity analysis of robustness, not as proof of it (Section 4). ICU, intensive care unit; IGF-1, insulin-like growth factor-1; IQR, interquartile range; LR, likelihood-ratio; REE, resting energy expenditure; SOFA, Sequential Organ Failure Assessment.

**Table 3:** Longitudinal trajectory of REE, SOFA and hormonal parameters across four ICU time-points.

Variable	Day 1	Days 5–7	Days 10–11	Days 13–14	LR χ^2^ (*p*)
REE, kcal/day	2051 ± 490 (*n* = 39)	2237 ± 498 (*n* = 28)	2378 ± 528 (*n* = 19)	2345 ± 500 (*n* = 16)	13.40 (0.004)
REE/kg, kcal/kg/d	25.3 ± 3.7 (*n* = 39)	26.8 ± 3.6 (*n* = 28)	27.2 ± 4.2 (*n* = 19)	26.8 ± 4.2 (*n* = 16)	17.24 (0.001)
SOFA score	6.3 ± 3.8 (*n* = 39)	7.5 ± 4.1 (*n* = 28)	7.4 ± 3.6 (*n* = 19)	7.3 ± 3.4 (*n* = 16)	3.86 (0.277)
Cortisol, μg/dL	21.1 ± 14.6 (*n* = 39)	17.3 ± 7.3 (*n* = 28)	16.6 ± 7.5 (*n* = 19)	13.4 ± 7.4 (*n* = 15)	7.76 (0.051)
IGF-1, ng/mL	80.0 ± 40.0 (*n* = 38)	90.4 ± 38.8 (*n* = 28)	91.3 ± 39.8 (*n* = 19)	105.4 ± 62.5 (*n* = 15)	7.42 (0.060)

Values are mean ± SD; *n* is the number of patients contributing data at each time-point (see Table 2 for causes of missingness). LR χ^2^ (*p*) is the likelihood-ratio statistic (3 df) for the overall time effect from a linear mixed-effects model with a random intercept per patient, using all available repeated measures. IGF-1, insulin-like growth factor-1; LR, likelihood-ratio; REE, resting energy expenditure; SD, standard deviation; SOFA, Sequential Organ Failure Assessment.

**Table 4:** Admission variables by ICU mortality status.

Admission Variable	ICU Survivors (*n* = 31)	ICU Non-Survivors (*n* = 8)	*p*
Age, years	54.0 (37.8–62.8)	66.0 (59.5–75.2)	0.013
REE, kcal/day	1928.5 (1745.8–2193.3)	2080.4 (1909.2–2237.4)	0.394
REE/kg, kcal/kg/day	24.8 (22.6–28.0)	24.3 (23.7–25.7)	0.972
SOFA score	6.0 (2.0–8.5)	9.0 (7.0–11.2)	0.012
Cortisol, μg/dL	18.7 (9.2–26.5)	24.5 (10.8–37.9)	0.384
IGF-1, ng/mL	78.0 (57.0–100.0)	67.0 (47.5–92.5)	0.498
Male sex, *n* (%)	21 (67.7%)	6 (75.0%)	1.000

Values are median (IQR) unless indicated. Comparisons used the Mann–Whitney U test (continuous) and Fisher’s exact test (categorical). Only admission (day-1) variables are shown; duration of mechanical ventilation and ICU length of stay are features of the clinical course and are omitted because their comparison by mortality status is confounded by in-ICU time at risk. *p*-values are descriptive. ICU, intensive care unit; IGF-1, insulin-like growth factor-1; IQR, interquartile range; REE, resting energy expenditure; SOFA, Sequential Organ Failure Assessment.

**Table 5:** Cox proportional hazards models for ICU mortality and univariable models for 28-day mortality (time variable: ICU length of stay).

Variable	HR (ICU)	95% CI	*p*	HR (28-Day)	95% CI	*p*
Age, per year	1.04	1.00–1.09	0.037	1.03	0.97–1.10	0.319
SOFA, per point	1.18	0.98–1.44	0.089	1.22	0.95–1.57	0.114
REE, per kcal/day	1.00	1.00–1.00	0.292	1.00	0.995–1.001	0.250
REE/kg, per kcal/kg/day	0.88	0.73–1.07	0.203	0.87	0.64–1.19	0.385
Cortisol, per μg/dL	1.01	0.96–1.05	0.815	1.00	0.94–1.06	0.908
IGF-1, per ng/mL	0.99	0.97–1.01	0.392	1.00	0.97–1.03	0.794
Multivariable: REE/kg	0.85	0.69–1.04	0.121	—	—	—
Multivariable: SOFA	1.25	1.00–1.57	0.055	—	—	—

All models use ICU length of stay as the time variable. ICU models: event = ICU death (8 events). 28-day models: event = death within 28 days (4 events); these are univariable only and are extremely imprecise. The multivariable model includes admission REE/kg and admission SOFA simultaneously. No hazard ratio is reported for duration of mechanical ventilation, which is collinear with the model’s time axis (ρ = 0.80) and therefore uninterpretable as an independent effect. *p*-values are descriptive and are not interpreted against a threshold. CI, confidence interval; HR, hazard ratio; ICU, intensive care unit; IGF-1, insulin-like growth factor-1; REE, resting energy expenditure; SOFA, Sequential Organ Failure Assessment.

**Table 6:** Associations of admission variables with ICU length of stay and duration of mechanical ventilation (*n* = 39), and adjustment of the REE–length-of-stay association for body size.

Admission Variable	ρ (ICU LOS)	*p*	ρ (MV Duration)	*p*
Age, years	−0.01	0.966	+0.07	0.656
REE, kcal/day	+0.55	<0.001	+0.47	0.003
REE/kg, kcal/kg/day	+0.25	0.121	+0.26	0.105
SOFA score	+0.43	0.006	+0.53	<0.001
Cortisol, μg/dL	+0.27	0.093	+0.17	0.298
IGF-1, ng/mL	+0.12	0.487	+0.04	0.788
Body mass index, kg/m^2^	+0.46	0.003	—	—
Fat-free mass, kg	+0.02	0.899	—	—
**Adjusted Model for ICU Length of Stay**		**REE Coefficient (β)**	***p***	
log(LOS) ~ admission REE + BMI		0.00088	0.026	
log(LOS) ~ admission REE + fat-free mass		0.00117	0.001	
REE per kg fat-free mass vs. LOS (Spearman ρ)		+0.56	<0.001	

ρ denotes the Spearman rank-correlation coefficient. ICU length of stay and duration of mechanical ventilation are downstream features of the clinical course and are strongly inter-correlated (ρ = 0.80); associations are interpreted in the forward direction only (admission variable → subsequent course). The lower panel shows that the association between absolute admission REE and ICU length of stay is not accounted for by body size. β is the coefficient for admission REE in a linear model of log-transformed ICU length of stay. BMI, body mass index; ICU, intensive care unit; IGF-1, insulin-like growth factor-1; LOS, length of stay; MV, mechanical ventilation; REE, resting energy expenditure; SOFA, Sequential Organ Failure Assessment.

## Data Availability

The data presented in this study are available on request from the corresponding author. The data are not publicly available due to privacy and ethical restrictions, as they contain potentially identifying clinical information from critically ill patients and the terms of the Institutional Review Board approval (467/11-1-2023) and the informed consent obtained from next of kin do not permit public deposition.

## Abbreviations

ACTH	Adrenocorticotropic Hormone
BMI	Body Mass Index
CI	Confidence Interval
CLIA	Chemiluminescent Immunoassay
ESPEN	European Society for Clinical Nutrition and Metabolism
FFM	Fat-Free Mass
HR	Hazard Ratio
ICC	Intraclass Correlation Coefficient
ICU	Intensive Care Unit
IGF-1	Insulin-like Growth Factor-1
IQR	Interquartile Range
LOS	Length of Stay
MAR	Missing At Random
MV	Mechanical Ventilation
REE	Resting Energy Expenditure
SD	Standard Deviation
SEM	Standard Error of the Mean
SOFA	Sequential Organ Failure Assessment
